# Amblyopia, Amaurosis, and the Extraction of Cataract

**Published:** 1866-10

**Authors:** Laurence Turnbull

**Affiliations:** Philadelphia


*Amblyopia, Amaurosis, and the Extraction of Cataract.* By LAURENCE TURNBULL, M. D., Philadelphia.

This translation was made during the past winter, for the Boston Medical and Surgical Journal, and is now collected from its pages. Its publication is stated by the translator for the twofold object of introducing Albrecht Von Græfe to the American medical public as a clinical teacher, and of exhibiting the progress which has been made in the exploration of one of the most obscure departments of ophthalmic science.

These lectures were compiled and reported by Dr. Engelhardt, and were translated from the *Klinische Monatsblœtter fur Augenheilkunde* for 1863 and '65. From our earliest attempts to master the subject of ophthalmology, these terms amblyopia and amaurosis have constantly stood in our way, as being arbitrary and used by all writers on diseases of the eye to express loss or defects of vision, and when the ophthalmoscope was brought into practical application, we trusted with others that these indefinite terms would pass away, and give place to terms of a more positive nature. But alas! the day has not yet come, for the recognized head of this department, as late as 1865, employs the same vague and uncertain terms, and thus writes and lectures upon them to students from every part of the world.

"In cases of amblyopia, (obscurity of vision—from which class we, of course, exclude all those affections which proceed from visible changes in the *refractive media*, or in the *internal structure* of the eye, as also cases of *neuroretinitis* and *embolia*) three things aid us in general in arriving at our conclusions. First, the *functional state* of the eye carefully considered; second, *the appearance of the papilla;* third, the *manner in which the affection* has become developed. Ordinary daylight is insufficient to detect slight defects in making a general examination of the periphery of the field of vision. This is to be conducted in a darkened room where the light proceeds from a graduated lamp, and a diaphragm being set at 100, and a black paper, without gloss, being held before the patient, (of course, at a fixed distance,) the limits of the field of vision are ascertained by means of white balls set on a black rod, and gradually removed from the point of fixation."

According to our author, the ophthalmoscope has determined that there are defined bounds to the class of amblyopic affections, by the exclusion of other intra-ocular diseases, and that there are four characteristic diagnostic points obtained by its use: *a*, alteration in color (of the optic nerve); *b*, opacity; *c*, excavation, and *d*, diminution of the calibre of the vessels. In the curative form, the papilla retains its delicate, semi-transparent color.

The following causes are given as producing this affection: The immoderate use of alcoholic liquors, frequent indulgence in strong cigars, pelvic obstructions, catamenial derangements, cold extremities, suppression of habitual hemorrhagic discharges, or of pathological or physiological secretions, venereal excesses, irregular sleep, and immoderate use of the eyes, sometimes exert a separate, sometimes a combined effect, and it is then difficult to assign each their part.

His treatment of a case of congestive amblyopia, with normal field of vision, is as follows: The use of alcoholic beverages must be given up, that of tobacco reduced to a minimum, regularity in diet and sleep. Local depletion, a rapid evacuation of blood by the leech of Heurtetoup, or by cupping in the neck. In hemorrhoidal affections, leeches to the anus, and in disorders of menstruation, cupping on the inside of the thighs. His diaphoretic treatment is principally (in imitation of many older practitioners) carried out by means of the decoction of Zittmann and the Roman or Turkish baths of dry heat instead of vapor. Abdominal disorders are treated by the use of the mineral springs of Marienbad, Kissingen, Homburg, and Carlsbad. Good and regular sleep must be had, and affords the most grateful refreshment to the active nerve of vision.

Having thus given what we consider the most interesting and instructive epitome of the author's views on the subject of amblyopia, we shall pass to the consideration of amaurosis. The course of the most desperate form is as follows:

"Slowly, but not regularly so, in the course of months or years, the field of vision of the first eye becomes contracted, (generally irregularly, laterally,) its acuteness of vision diminishes, atrophic degeneration of the papilla takes place, and the organ is lost, while after the first eye has began to be affected, sometimes not till after its entire loss, the second commences to run the same course. These cases are indeed utterly hopeless; they are regarded as a *noli me tangere* by the experienced physician, who cautiously refrains from active treatment, knowing that this may easily harm, and at the best can be of little service.

A few remarks may be added, on the nature of amaurosis. Where there are no objective intraocular symptoms in cases of impaired vision, we are apt to speak of cerebral or spinal amaurosis. Especially interesting is the connection of progressive amaurosis with paralysis and mental alienation—the course of the degeneration is from the vertebral column toward the interior of the skull. Of all the many cases of spinal amaurosis, (forming as they do, some thirty per cent. of the graver forms of progressive amaurosis,) which has come under Von Græfe's observation, he can recollect but two instances where the disease progressed in an opposite direction. It may be regarded as a fixed fact that there is no question of an inflammation of the nerve connective tissue, in the ordinary sense of the word, in amaurosis or tubes dorsalis. By the ophthalmoscope it is found that in the optic papilla there is either simple loss of substance, (atrophic excavation,) the most perfect type of a true atrophic process; sometimes there occurs a gradual consolidation of the connective tissue, the papilla growing smooth, white, and opaque, while the lamina cribrosa becomes hidden.

Having given his treatment of a case of congestive amblyopia, we shall now give an abstract of his treatment of a case of progressive amaurosis, depending on atrophy of the optic nerve.

It is simply necessary to state that all powerful derivative agents, cathartics, seatons, mercurials, diaphoretics, and depletives, beneficial as they may be in cases of congestive amblyopia, (Case 1,) are here decidedly injurious. In the average of cases of progressive atrophy, the best means of retarding its progress consists in a mild tonic course—small doses of an iron salt, and tonic baths, milk and whey diet; in other words, a nutritious, but not stimulating diet, good air, a moderate course of cold bathing, and carefully regulated amount of light. Cases 3 to 8 are interesting and also instructive, and are well worthy of perusal, but our want of space prevents us from entering even upon an abstract. In concluding this part of our subject, we may state that the terms amaurosis and amblyopia are merely symptoms of diseased eyes. The true typical symptoms are a diminution in the acuity of vision, (*s*) and should be carefully investigated by test types, optometer, by concave and convex glasses, and the ophthalmoscope.

[TO BE CONTINUED.]

